# Incorporating Social Determinants of Health in Infectious Disease Models: A Systematic Review of Guidelines

**DOI:** 10.1177/0272989X241280611

**Published:** 2024-09-21

**Authors:** Shehzad Ali, Zhe Li, Nasheed Moqueet, Seyed M. Moghadas, Alison P. Galvani, Lisa A. Cooper, Saverio Stranges, Margaret Haworth-Brockman, Andrew D. Pinto, Miqdad Asaria, David Champredon, Darren Hamilton, Marc Moulin, Ava A. John-Baptiste

**Affiliations:** Department of Epidemiology & Biostatistics, Schulich School of Medicine and Dentistry, Western University, London, ON, Canada; Centre for Medical Evidence, Decision Integrity & Clinical Impact (MEDICI), Schulich School of Medicine and Dentistry, Western University, London, ON, Canada; Schulich Interfaculty Program in Public Health, Western University, London, ON, Canada; Department of Epidemiology & Biostatistics, Schulich School of Medicine and Dentistry, Western University, London, ON, Canada; University of Ottawa Heart Institute, Ottawa, ON, Canada; Public Health Agency of Canada, Ottawa, ON, Canada; Agent-Based Modelling Laboratory, York University, Toronto, ON, Canada; Center for Infectious Disease Modeling and Analysis, Yale School of Public Health, New Haven, CT, USA; Department of Medicine, Johns Hopkins University School of Medicine, USA; Department of Health, Behavior, and Society, Johns Hopkins Bloomberg School of Public Health, USA; Department of Epidemiology & Biostatistics, Schulich School of Medicine and Dentistry, Western University, London, ON, Canada; Department of Clinical Medicine and Surgery, University of Naples Federico II, Italy; Department of Sociology, University of Winnipeg, MB, Canada and National Collaborating Centre for Infectious Diseases, Winnipeg, MB, Canada; Division of Clinical Public Health, Dalla Lana School of Public Health, University of Toronto, Toronto, ON, Canada and Department of Family and Community Medicine, Faculty of Medicine, University of Toronto, Toronto, Ontario, Canada; Department of Health Policy, London School of Economics and Political Science, UK; Public Health Agency of Canada, National Microbiological Laboratory, Guelph, ON, Canada; London Health Sciences Centre, London, ON, Canada; London Health Sciences Centre, London, ON, Canada; Department of Anesthesia & Perioperative Medicine, Schulich School of Medicine and Dentistry, Western University, London, ON, Canada; Department of Epidemiology & Biostatistics, Schulich School of Medicine and Dentistry, Western University, London, ON, Canada; Centre for Medical Evidence, Decision Integrity & Clinical Impact (MEDICI), Schulich School of Medicine and Dentistry, Western University, London, ON, Canada; Schulich Interfaculty Program in Public Health, Western University, London, ON, Canada; Department of Anesthesia & Perioperative Medicine, Schulich School of Medicine and Dentistry, Western University, London, ON, Canada

**Keywords:** social determinants of health, infectious disease models, modelling guidelines, equity, pandemic

## Abstract

**Background:**

Infectious disease (ID) models have been the backbone of policy decisions during the COVID-19 pandemic. However, models often overlook variation in disease risk, health burden, and policy impact across social groups. Nonetheless, social determinants are becoming increasingly recognized as fundamental to the success of control strategies overall and to the mitigation of disparities.

**Methods:**

To underscore the importance of considering social heterogeneity in epidemiological modeling, we systematically reviewed ID modeling guidelines to identify reasons and recommendations for incorporating social determinants of health into models in relation to the conceptualization, implementation, and interpretations of models.

**Results:**

After identifying 1,372 citations, we found 19 guidelines, of which 14 directly referenced at least 1 social determinant. Age (*n* = 11), sex and gender (*n* = 5), and socioeconomic status (*n* = 5) were the most commonly discussed social determinants. Specific recommendations were identified to consider social determinants to 1) improve the predictive accuracy of models, 2) understand heterogeneity of disease burden and policy impact, 3) contextualize decision making, 4) address inequalities, and 5) assess implementation challenges.

**Conclusion:**

This study can support modelers and policy makers in taking into account social heterogeneity, to consider the distributional impact of infectious disease outbreaks across social groups as well as to tailor approaches to improve equitable access to prevention, diagnostics, and therapeutics.

**Highlights:**

- Infectious disease (ID) models often overlook the role of social determinants of health (SDH) in understanding variation in disease risk, health burden, and policy impact across social groups.
- In this study, we systematically review ID guidelines and identify key areas to consider SDH in relation to the conceptualization, implementation, and interpretations of models.
- We identify specific recommendations to consider SDH to improve model accuracy, understand heterogeneity, estimate policy impact, address inequalities, and assess implementation challenges.

## Background

Infectious diseases (IDs) tend to have a disproportionate impact on underserved communities.^1–7^ This was recently witnessed during the COVID-19 pandemic, which has disproportionately affected underrepresented racial groups, deprived socioeconomic groups and the elderly, both through its unequal health burden and disparity of economic losses.^1,2,8–15^ Evidence on distributional disparities emerged early in the pandemic when a number of studies showed elevated COVID-19 mortality risk among Black Americans.^2,8,9,13,16–18^ Similar data from other countries showed that hospitalization and mortality rates were inversely correlated with socioeconomic status.^19–24^ These disparities are a symptom of deeper systematic societal and health care inequities, including disproportionate exposure through high-risk employment, prevalence of comorbidities, and inequitable access to testing and treatment.^3,9,11,25–27^

Throughout the pandemic, ID models have informed policy decisions at local, national, and global levels. These models have been used extensively to make predictions about the course of the pandemic and forecast spatiotemporal epidemiological trends.^28–35^ In many cases, regions and countries made major policy shifts informed by model-based evidence. Models have estimated the impact of macro-level decision scenarios, including lockdowns and other public policy decisions,^36–40^ compared testing and disease control policies in the community and workplaces,^39,41,42^ predicted the impact of vaccination on the pandemic,^43–46^ and estimated the burden on health services.^47–49^

Despite empirical evidence on significant inequalities in the impact of the pandemic, ID models, particularly those developed early in the pandemic, were mostly based on an average population approach without accounting for heterogeneity in risk and the disproportionate impact of the pandemic on social groups.^50^ This may have resulted largely from the past practice of modeling but also, in part, from a lack of guidance on how ID models should incorporate social determinants of health.

## Purpose

To underscore the importance of considering social heterogeneity in ID modeling, we conducted a systematic review of existing guidelines for developing and reporting ID models. The aim of this review is to identify recommendations for incorporating social determinants of health into ID models in relation to the motivation, conceptualization, implementation, and interpretations of such models. To situate the recommendations identified in this review in the context of the current modeling practices, we interpret them in relation to the recent COVID-19 pandemic and ongoing epidemics (e.g., HIV, tuberculosis) as relevant. Since the pandemic catalyzed the development of a large number of models, leveraging an unprecedented allocation of resources, expertise, and international collaboration, it particularly offers insight into interpreting modeling recommendations.

## Methods

This systematic review is reported according to the Preferred Reporting Items for Systematic Reviews and Meta Analyses (PRISMA) guidelines.^51^ The protocol was registered in PROSPERO (CRD42021231097).^52^

### Data Sources and Study Selection

We searched the Medline, Embase, Cochrane Library, and Web of Science databases for ID modeling guidelines published between January 2000 and January 1, 2022. The modeling search terms included the following: “model” adjacent to “infectious disease,” “compartmental,” “individual-based,” “agent-based,” “network,” “Markov chain or process,” “Monte Carlo method,” “dynamic,” “simulation,” and “mathematical.” These were combined with infectious disease terms including “communicable diseases,” “transmission,” “outbreak,” “epidemic or pandemic,” and guidelines terms. The full search strategy is presented in Supplemental Table S1.

We included publications that were labeled guidelines by the authors or included recommendations or strategies for developing ID models. Guidelines addressing mathematical or statistical techniques (e.g., mechanistic, mathematical, network, simulation, autoregressive, Markov, or other model types) to characterize the epidemiology or the direct and/or indirect impact of infectious diseases at the individual, population, health/non–health systems levels were included. We also manually searched the references in the identified studies. There was no restriction on the type of infectious disease or the mode of transmission of the infection. We excluded clinical guidelines that focused only on screening, disease management, or population interventions for infectious diseases, without any reference to modeling. Specific inclusion and exclusion criteria are listed in Table 1.

**Table 1:** Inclusion and Exclusion Criteria Used to Identify Infectious Disease Modeling Guidelines

Inclusion Criteria	Exclusion Criteria
1. Guidelines focusing on modeling infectious disease in general 2. Guidelines focusing on modeling specific infectious diseases (such as COVID-19, influenza, tuberculosis), including diseases transmitted to humans by nonhumans 3. Guidelines focusing on the epidemiology or the direct and/or indirect health or nonhealth impact of infectious diseases at the level of individuals, population, and/or health or nonhealth systems 4. Guidelines should include recommendations or strategies to develop mathematical or statistical models of infectious diseases	1. Animal models or animal version of human pathogen 2. Qualitative and behavioral studies 3. In vitro, pharmacodynamic or pharmacokinetic studies 4. Focus on chronic diseases whose main cause is not infectious, e.g., diabetes modeling guidelines 5. Guidelines on screening, clinical management, or population interventions for infectious diseases only, without focus on modeling 6. Non-English publication language

### Main Outcome and Social Determinants

The main outcome of this systematic review was whether or not ID modeling guidelines mentioned the need for incorporating social determinants of health and provided reasons and/or recommendations to do this in relation to the conceptualization, implementation, and interpretations of ID models. Importantly, for modeling guidelines that discussed social determinants, the reasons and recommendations were identified and summarized.

To identify social determinants of health in guidelines, we used the World Health Organization definition to include any social factor related to the conditions “in which people are born, grow, work, live, and age.”^53^ We acknowledge that there is variation across national and international agencies on the list of social factors considered to be social determinants. For this study, we used an inclusive approach and considered the following social determinants (and related concepts): age, sex, gender, sexuality, race, ethnicity, immigrant status, culture, religion, education, childhood experiences, biological/genetic endowment, socioeconomic status, social protection, employment and working conditions, food security, social inclusion and support, housing and basic amenities, physical environment and geography, and access to affordable health services. We note that some of these factors, for example, age and sex, are often considered only as demographic factors in modeling studies; however, there is growing recognition of the social and institutional influence of these factors on health outcomes. For instance, the COVID-19 pandemic has exposed age-related inequities in access of health services, lack of protection in care homes, economic vulnerability, and the disparate impact of public health policies on social isolation and mental health of the young and old age groups.^54,55^

### Data Extraction, Quality Assessment, and Synthesis

The titles and abstracts of the studies retrieved using the search strategy were screened independently by 2 reviewers (Z.L. and N.M.) to identify studies that potentially met the inclusion criteria. Conflicts were resolved by two independent reviewers (S.A. and A.J.-B.). Full texts of potentially eligible studies were retrieved and independently assessed for eligibility by S.A. and A.J.-B. Data extraction was independently conducted by 2 reviewers (Z.L. and N.M.). From each of the included publications we abstracted information on the publication source, year, specific IDs targeted (where applicable), and the aspects of modeling addressed in the guidelines, including conceptualization, implementation, validation, or calibration and reporting. We assessed whether the guideline mentioned the need to incorporate 1 or more social determinants of health into ID models. This may have been addressed directly or indirectly, with the former meaning that the guidelines discussed specific social determinants and/or methods to include them in models, and the latter indicating that the guidelines acknowledged social heterogeneity of outcomes without discussing specific social determinants and making recommendations. We extracted data on the rationale for incorporating social determinants and specific recommendations on when and how to incorporate them into models.

Following this, 2 reviewers (Z.L. and N.M.) independently carried out the thematic analysis of recommendations and grouped them into categories, in consultation with study leads (S.A. and A.J.-B.). Specifically, we followed a 6-phase analytical approach recommended by Braun and Clarke^56,57^; this involved familiarization with the data (i.e., the recommendations in the ID guidelines), generating initial codes, generating themes, reviewing potential themes, defining and naming themes, and reporting the findings. We used an inductive approach; that is, we developed an open coding framework, instead of fitting recommendations to predefined coding categories. Following this, we evaluated the extracted recommendations based on their content and context and identified common patterns across codes to group them into meaningful and systematic themes, an approach referred to as “semantic coding.” This thematic categorization was shared with the study team to reach consensus through an iterative process. A similar approach has been followed in previous studies.^58,59^ Finally, recommendations under each theme were summarized and, where relevant, supported by insights from the recent pandemic.

We used the international Appraisal of Guidelines, Research and Evaluation v. II (AGREE II) checklist to assess the methodological quality and transparency of the identified guidelines.^60,61^ While AGREE II was originally developed to assess the quality of clinical practice guidelines, most items in the checklist are relevant to all guidelines, including modeling. We are not aware of any quality checklists that are specific to modeling guidelines. AGREE II includes 23 items in 6 domains: Scope and Purpose, Stakeholder Involvement, Rigor of Development, Clarity of Presentation, Acceptability, and Editorial Independence. Authors of AGREE II allow adaptation of the checklist to specific context; we therefore excluded 11 items (items 2, 3, 5, 7–9, 11, 12, 14, 16, 21) that were not applicable to modeling guidelines.

## Results

### Identification of Studies

**Figure 1:**
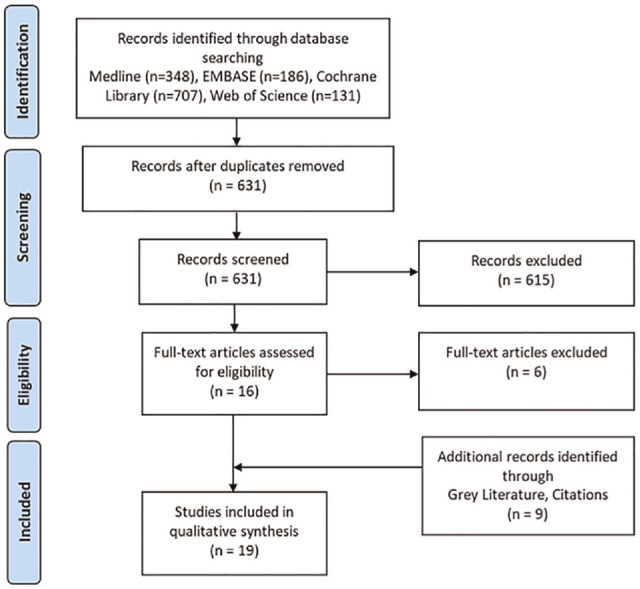
PRISMA diagram of study selection and screening.

Our literature search identified a total of 1,372 records: Medline (348), EMBASE (186), Cochrane Library (707), and Web of Science (131). A total of 741 duplicate records were removed, and the titles and abstracts of 631 studies were screened, of which 615 records were excluded. Sixteen peer-reviewed full-text articles were assessed for full eligibility, and 10 were retained. An additional 9 studies were identified through gray literature search and through a manual search of reference lists. In total, 19 studies were included for data extraction. The PRISMA diagram is presented in Figure 1.

The full list of studies is presented in Supplement S2. Thirteen of 19 guidelines focused on general/nonspecific ID modeling, whereas 6 were on specific IDs or group of diseases; these included 1 study each on COVID-19, HIV, influenza, Chagas disease, neglected tropical diseases, and zoonotic diseases.

### Social Determinants Identified in Guidelines

**Figure 2:**
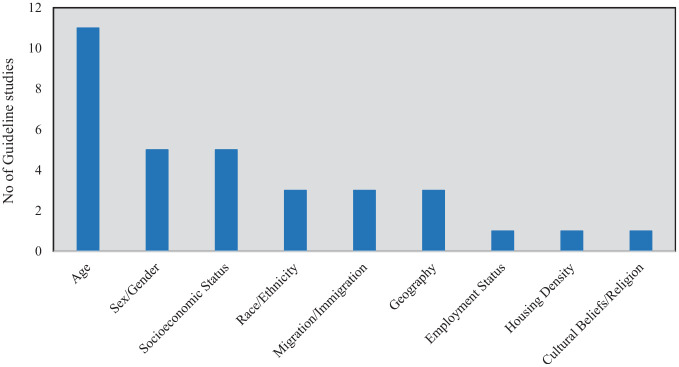
Social determinants mentioned in guidelines for incorporation into infectious disease models.

Of the 19 guidelines identified, 14 made direct and specific reference to at least 1 social determinant and made recommendations to consider them in models (Figure 2). Age was the most common factor across all guidelines (11 studies); however, age is often discussed in guidelines only as a demographic factor and not as a social determinant of health. Age was followed by sex/gender (5 studies), socioeconomic status, deprivation or marginalization (5 studies), race or ethnicity (3 studies), immigration or migration patterns (3 studies), geography, urbanization and rurality (3 studies), housing density (1 study), employment status (1 study), and cultural beliefs and religion (1 study). Four of 19 studies made general reference to social determinants of health (without discussing specific factors). These studies suggested that models should consider population demography, rurality, setting, and the level of access to services to address diversity of decision contexts, evaluate interventions in different settings, and appraise implementation strategies.

### Focus of Included Guidelines

Table 2 identifies the aspect of modeling covered by each guideline. All guidelines focused on conceptualization of the modeling approach, and most also addressed the relevance of social determinants in this context. Model calibration and/or validation were addressed by most guidelines while reporting of the findings of modeling studies was less frequently addressed. Three guidelines (Knight 2016, Russell 2017, and COVID-19 CMCC Policy Group 2020) provided general frameworks and principles for ID modeling. Three (Ultsch 2016; CGCDM 2019; Moghadas 2015) presented consensus statements from workshops on ID modeling methods. Seven (Jit 2011; Caro 2012; Pitman 2012; Roberts 2012; Moghadas 2015; Funk 2015; den Boon 2019) stated best practice guidelines for ID modeling. Moghadas 2015 and Funk 2015 discussed the challenges of ID modeling and provided consensus recommendations. Barnes (2014) included a checklist to evaluate ID models, while Caro (2014) included a questionnaire to evaluate models. COVID-19 CMCC is the only study focusing on COVID modeling.

**Table 2:** Aspects of Modeling Addressed by the Identified Guidelines

			Which Aspects of Modeling Did the Guideline Address?			
Study	Focus of the Guideline (i.e., Framework)	Social Factors Discussed in the Guideline	Conceptualization (Y/N)	Implementation (Y/N)	Validation/ Calibration (Y/N)	Reporting (Y/N)
Abuelezam 2013	Reporting guidelines	Age; migration	Y	Y	Y	Y
Andradóttir 2014	Review	Age, employment status	Y	Y	Y	N
Barnes 2016	Checklist	Age, gender, and race	Y	Y	Y	Y
Behrend 2020	Principles	No specific factor	Y	N	Y	Y
Caro 2012	Best practice guidelines	No specific factor	Y	Y	Y	Y
Caro 2014	Questionnaire	Age, sex, nationality, race/ethnicity	Y	Y	Y	Y
CGCDM 2019	Consensus statement from workshop	No specific factor	Y	Y	Y	N
CMCC Policy Group 2020	Framework	Age, socioeconomic status, housing density, migration patterns	Y	Y	Y	Y
den Boon 2019	Guidelines	No specific factor	Y	Y	Y	Y
Drake 2018	Principles and guidelines	Age, sex, socioeconomic status	Y	N	N	Y
Funk 2015	General guidelines	Socioeconomic status, cultural beliefs, and religion	Y	Y	N	N
Jit 2011	Guidelines	Age	Y	Y	N	N
Knight 2016	Framework	Immigration	Y	Y	N	N
Moghadas 2015	Consensus statement from workshop or best practice guidelines	Sex, race/ethnicity, socioeconomic status, geography	Y	Y	Y	Y
Pitman 2012	Best practice guidelines	Geography, socioeconomic status and genetics	Y	Y	Y	Y
Roberts 2012	Best practice guidelines	Age	Y	Y	Y	Y
Russell 2017	Framework	Age	Y	Y	Y	N
Ultsch 2016	Consensus framework	Age	Y	Y	Y	Y
Woolhouse 2011	Review	Age, sex, population density, urbanization	Y	Y	Y	Y

In relation to model conceptualization and implementation, Barnes (2016), Pitman (2012), and Woolhouse (2011) recommend considering social heterogeneity in age, sex, gender, and race/ethnicity in the underlying risk distribution, prevalence, transmission routes, spatial distribution, and mixing patterns. In addition, heterogeneity in behavioral responses to public health interventions (such as lockdowns) is crucial at both the conceptual and implementation stages to accurately predict changes in the frequency and type of interactions and adherence to public health guidelines (Andradóttir 2014; Jit 2011; CMCC 2020). Funk (2015) and Knight (2016) emphasize that behavioral practices should be considered during model development, in relation to cultural and religious beliefs and norms, historical patterns, and local choices that may be spatially clustered. Model implementation should also account for differences in socioeconomic status and family structures, particularly when considering disease-driven migration patterns during an epidemic/pandemic (Funk 2015). Other important considerations include geography, population density, living conditions (such as refugee camps), climate (Pitman 2012; CMCC 2020), and occupation (Jit 2011). Immigration may be a relevant contextual factor for some IDs, such as tuberculosis (Knight 2016).

In relation to model calibration and validation, Andradóttir (2014) and Caro (2014) stress that social determinants, including age, sex, and race/ethnicity, should be considered to ensure that the characteristics and statistical properties of the modeled population match the observed data. National models adapted for subnational predictions should be calibrated and validated using local population data, reflecting sociodemographic distribution and inequities as well as heterogeneity in health system capacity, preparedness, and access to services (CMCC 2020). Such calibration may require local demographic data and sociocultural context to incorporate unique population characteristics. Finally, sociodemographic factors are also relevant to the reporting of model outcomes, particularly when multiple intervention scenarios are being evaluated (Drake 2018). Reporting should be disaggregated by sociodemographic groups to reflect differences in the disease course, intervention impact, costs, and health outcomes (Roberts 2012). This is particularly important when considering inequities in relation to risk heterogeneity and/or program design and benefits (Drake 2018).

### Quality Appraisal of Included Studies

The median scores for individual domains are presented in Supplemental Table S1. The highest median scores were in the “Clarity of Presentation” domain (median 91.7%; range 54.2%–95.8%) and the “Editorial Independence” domain (median 87.5%; range 45.8%–95.8%), as most guidelines clearly highlighted recommendations and managed conflicts of interest. The lowest median score was for the “Rigor of Development” domain (median 54.2%; range 16.7%–87.5%), indicating low quality overall for the reported guideline development methodology. The mean score for “Scope and Purpose” was 83.3% (range 66.7%–100%), “Stakeholder Involvement” was 70.8% (range 25%–95.8%), and “Applicability” was 86.1% (range 58.3%–94.4%).

We also assessed the potential association between the quality of the guidelines and whether or not social determinants were considered. However, we did not find a clear relationship. More specifically, 8 guidelines (Andradottir 2014; Barnes 2016; Funk 2015; Drake 2018; Knight 2016; Moghadas 2015; Russell 2017; CMCC 2020) had a low score of <50% on domain 3 (i.e., Rigor of Development) but all 8 had discussed at least 2 social factors in the guidelines. Only 1 study (Jit et al 2011) scored low on domain 2 (i.e., Stakeholder Involvement) but included age as a social factor to consider in ID models. Finally, only 1 study (Caro et al. 2012) scored low on domain 6 (i.e., Editorial Independence) and did not explicitly discuss any social factor in the guideline. All other quality scores across all studies were ≥50%. We also investigated if the quality of the guidelines was associated with the aspects of modeling addressed in the identified guidelines (as in Table 2) but did not find any clear association.

### Recommendations for Considering Social Determinants of Health

The reasons and recommendations for considering social determinants in ID modeling varied significantly across guidelines. We grouped these into 5 categories and, where relevant, discuss their relevance to the current pandemic.

#### 1. Incorporating social determinants can improve predictive accuracy of models

The linear and nonlinear interplay of social determinants can significantly influence the spread of IDs (Woolhouse, 2011). Ten studies (Woolhouse, 2011; Pitman, 2012; Ultsch, 2012; Abuelezam, 2013; Andradóttir, 2014; Andradóttir, 2014; Funk, 2015; Caro, 2014; Knight, 2016; CMCC, 2020) highlighted that incorporating social determinants of health to appropriately characterize and parametrize the disease dynamics can improve model accuracy, precision, and validity. For instance, for models of HIV transmission, social determinants such as age, gender, socioeconomic status, migration, and cultural context may determine the level of sexual contact, which influences the level of transmission (Abuelezam 2013). Similarly, population age structure and multimorbidity distribution are critical risk factors for predicting hospitalization and mortality rates, as seen in COVID-19 models (CMCC 2020). In developing countries, population density in urban slums and refugee camps is an important predictor of infection rate.^62^ In short, the choice of data inputs and parameters related to social groups can affect model outcomes/outputs, determine how well the model mimics the real world, and influence model quality, plausibility, and generalizability.

#### 2. Understanding heterogeneity of disease burden and policy impact

##### Heterogeneity of disease burden

Fifteen studies (Funk 2015; Barnes 2016; Caro 2012; den Boon 2019; Ultsch 2016; Woolhouse 2011; Andradóttir 2014; CGCDM 2019; CMCC 2020; Jit 2011; Roberts 2012; Behrend 2020; Abuelezam 2013; Caro 2014; Pitman 2012) recommended that health burden should be modeled by social groups due to heterogeneity in disease risk based on social, housing, and occupational circumstances; level of interaction and movement of population groups; underlying clinical risk; and behavioral adaptation to epidemics/pandemics. Also, social groups with high risk may be clustered spatially, leading to geographical heterogeneity and high-incidence hot spots (Funk 2015). Modeling disease burden in specific population groups requires knowledge of host demography and movement patterns (Woolhouse 2011; Andradóttir 2014; Ultsch 2016; Pitman 2012; CMCC 2020). During the COVID-19 pandemic, significant social heterogeneity was observed, including higher risk in ethnic minorities (e.g., African American population), socioeconomically disadvantaged groups, and the elderly, particularly the residents of long-term care homes.^30^ Average population models may overlook this heterogeneity in disease impact across groups.

##### Heterogeneity of policy impact

Decision modelers should consider the possibility of heterogeneous response to policies or interventions across population groups (Jit 2011). For instance, the impact of a physical distancing policy on infection control, hospitalization, and mortality rate may vary across population groups based on socioeconomic status, occupation (e.g., flexible remote work v. in-person jobs), rural/urban geography, spatial distribution, and access to services (Woolhouse 2011). Access to health services and behavioral responses to the pandemic, such as personal decisions about testing and vaccination and the level of adherence to public health orders, may also differ between social groups, which can in turn influence the impact of policies across social groups (Funk 2015). For instance, the stay-at-home order during the COVID-19 pandemic did not reduce the work-related risk of infection in low-wage earners and essential workers at the same level.^63^ This impact disparity was worse in developing countries with limited access to personal protective equipment at work and no public funds for informal workers, as these workers must go out to earn a living and face the risk of infection.^64^ Models that use a population-averaged approach do not account for impact heterogeneity and in turn may over- or underestimate the effectiveness of a policy for certain social groups and can mislead decision makers (CMCC 2020; Jit et al. 2011).

Identifying population groups that may vary in terms of the underlying social circumstances and behavioral responses is a good starting point for modeling the impact of heterogeneity (den Boon 2019; Funk 2015; Caro 2012; Behrend 2020; Abuelezam 2013; Caro 2014). den Boon (2019) recommended that analysts should separately modify model parameters to identify if disease policy varies by social groups. Funk (2015) recommended that analysts formulate a collection of models incorporating different aspects of social circumstances and behavioral responses as input parameters and then use model selection methods to determine the most appropriate approach in which the model best fits the data.

#### 3. Contextualizing decision making

Twelve studies (Roberts 2012; Knight 2016; Caro 2014; Caro 2012; den Boon 2019; Pitman 2012; Ultsch 2016; Andradóttir 2014; Drake 2018; Jit 2011; CMCC 2020; Woolhouse 2011) highlighted the significance of considering social determinants in relation to decision making. This implies not only that modelers should explore social heterogeneity at the stage of analysis but also that the decision problem itself should be conceptualized and contextualized in relation to social determinants. Following this, models can be developed, calibrated, and evaluated to reflect social distributions as they relate to decision scenarios (Andradóttir 2014; den Boon 2019). This is important because models are meant to support real-world decision making, which occurs within a social setting (Caro 2012; Knight 2016).

Caro (2014) recommended that analysts should consider the decision context of the model in relation to the sociodemographic structure, social behavior, health system characteristics, and geographical distribution of social groups. Models that involve decision making should clearly describe policy options in relation to the expected variation in implementation, uptake, and/or effectiveness across different subgroups. Modifying implementation strategies, such as varying intervention eligibility, availability, coverage, or policy compliance, may be needed to address specific decision goals for social groups (den Boon 2019). Four studies (den Boon 2019; Pitman 2012; Woolhouse 2011; Abuelezam 2013) proposed performing sensitivity and scenario analyses to evaluate the impact of decision options across social groups. Finally, when applying the results of ID models, decision makers should consider the social context, such as cultural and religious beliefs and historical context, to inform implementation decisions (Knight 2016).

#### 4. Considering inequalities

Six studies (Ultsch 2016; Moghadas 2015; Drake 2018; CMCC 2020; Jit 2011; Roberts 2012) underlined that incorporating social determinants of health in ID models is critical for understanding and evaluating the equity impact of IDs. Here, equity, as opposed to variation in health, implies underlying unfairness in disease risk, burden, and policy impact. Differences can be expected across social determinants of health, including socioeconomic and racial groups, Indigenous/non-Indigenous identities, sex, gender, and age (Moghadas 2015). Integrating health equity considerations requires modelers to apply the principle of fairness, accounting for structural differences between different social groups (Drake 2018). The report by Drake (2018) noted that “equity heterogeneity can be correlated with or create risk-heterogeneity,” and “risk-heterogeneity could contribute to equity variation.” Models that incorporate social determinants with an equity lens can assist policy makers in identifying social gradient in health burden and examining the impact of a policy across different social groups (Moghadas 2015; Drake 2018). In addition, findings from infectious models can support the design of tailored public health interventions to reduce health inequity (Knight 2016). Models ignoring this social variation may lead to decisions that exacerbate health inequities.

Moghadas (2015) recommended that modelers should work with stakeholders to formulate research questions, ensuring that decision context and input parameters reflect the values and preferences of different sectors of society. This requires forging links with community groups and developing collaborative networks, such as “community of practice,” to guide equity-informed public health response.^65,66^ For models that make predictions concerning vulnerable populations, modelers should consider differential health status and risk factors driven by inequity, such as migration, testing capacity, and access to health care. Accordingly, Moghadas (2015) proposed developing a model framework to provide better guidance on assessing health inequities.

Overall, while a few guidelines have offered recommendations for considering social determinants of health in the context of health inequalities, these are mostly discussed as broad principles. Moreover, we observed notable variation among guidelines regarding the social determinants of health domains to consider their relevance to health inequalities. Despite their inadequacy, these recommendations provide a motivating context for equity-informative ID models. However, there is still a need for clearer recommendations on how to conceptualize, contextualize, and operationalize considerations of inequality within these models.

#### 5. Technical implementation considerations

Ten studies (Russell 2017; Pitman 2012; Funk 2015; Roberts 2012; Andradóttir 2014; Abuelezam 2013; Behrend 2020; Jit 2011; Ultsch 2016; Woolhouse 2011) discussed technical recommendations for incorporating social determinants in ID models. These include identifying the target population to consider population heterogeneity, performing subgroup analysis based on different transmission characteristics to evaluate the impact of public health interventions, and conducting sensitivity analysis to examine different model parameters.

A key consideration is the choice of modeling framework, which ranges from compartmental models to more complex individual-based and network models. Several guidelines noted that commonly used compartmental models (such as the susceptible-infected-recovered models) typically assume homogeneity of the population within each compartment and may not appropriately reflect variation in the impact of the pandemic across social groups and geographic region (Russell 2017). Moving to individual-level models can be challenging as they tend to be more complex and “data hungry” and require greater computational power and time; however, individual-level models may be more appropriate for incorporating social determinants of health while improving precision and accuracy of prediction. However, one should note that increasing complexity does not automatically imply that the model is better; less complex models can also be used to represent social heterogeneity and equity considerations.^67^ The simplest step would be to extend the commonly used compartmental models by stratifying compartments by relevant social groups.^67^

Another technical consideration addressed by guidelines relates to the data used to inform model parameters. Ultsch (2016) recommended that modelers use empirical data from surveys, disease surveillance studies, and administrative and demographic data sources to derive contact matrices in specific populations. However, challenges related to availability, completeness, and accuracy of personal identify data, such as race and ethnicity, in health administrative and surveillance data are well documented in the literature.^68,69^ Moreover, microdata are often not representative, and any biases in data would then translate to biases in model predictions that may inequitably and adversely affect some social groups. For instance, surveillance and survey data may not be sufficient for estimating infection rates and transmissibility because some social groups may be underrepresented due to unequal utilization or access to the health system.^67^ As a result, disease prevalence may be underestimated and disease severity and health outcomes may be overestimated in some social groups (Pitman 2012). Pitman (2012) proposed using seroprevalence curves categorized by age, sex, and other relevant factors to estimate disease transmissibility. Estimates derived using synthetic methods should be validated against survey data because synthesized data from multiple studies may include individuals from heterogeneous social groups (Ultsch 2016).

## Discussion

ID models have taken the center stage in informing decision making during previous epidemics and the current COVID-19 pandemic. A large majority of these models use a population-averaged approach, which treats the population as a homogenous group in relation to disease risk and transmission. However, the COVID-19 pandemic has drawn attention to the significance of social determinants in relation to the burden of diseases and the impact of policies. This motivated our review of ID guidelines to understand the scope and recommendations for incorporating social determinants of health in ID models. After searching 4 large databases, 19 eligible guidelines were identified. Most (13/19; 68%) studies were general guidelines applied to all IDs, and the remaining focused on specific IDs (such as HIV). Guidelines suggest that ID models that incorporate social determinants can better characterize disease dynamics, improve model accuracy, understand heterogeneity of disease risk and policy impact, contextualize decision making, and evaluate inequities.^70–73^ Age was the most commonly discussed social determinant of health incorporated in models to reflect the demographic structure and risk in the population; this was followed by sex/gender and socioeconomic status. Which social determinants are relevant may depend on the ID being modeled. For instance, age, culture, and education are important factors for modeling HIV transmission, while age, racial/ethnicity, socioeconomic status, population density, occupation, and institutional residence are highly relevant for modeling respiratory infections such as SARS-CoV-2. Some social determinants, such as age, were discussed as demographic factors in modeling guidelines; however, the relevance of their social and institutional context has become clearer during the current pandemic.

In relation to the list of social determinants considered in this review, the following factors were not explicitly mentioned in the identified studies: sexuality, social inclusion, social protection, and physical environment. Depending on the disease being modeled, these factors may have an important influence on predicting disease risk and transmission. For instance, the multilayered physical and social context of the built environment,^74,75^ social networks and community vulnerability,^76^ and sexual orientation^77^ may be relevant to different degrees for specific ID models. In some cases, these relationships may be complex and require a deeper understanding of individual behaviors along the course of the epidemic.^78^

This systematic review also identified several methodological challenges in incorporating social determinants of health into ID models. Limited data to inform parameter values for variation in disease risk, exposure, behavioral response, and access to health services by social determinants represents a key challenge. Synthetic methods have been proposed to derive parameters by age, sex, and socioeconomic status using survey data; however, data sources are rarely sufficient to comprehensively estimate for all relevant parameters, particularly those related to sociocultural and behavioral determinants of health.^79^ Guidelines also highlighted that social heterogeneity of risk and disease dynamics are better captured using stochastic individual-based models; however, the computational complexity of these models and the need for detailed data can prohibit the widespread use of these models.^80^ The model choice may be an empirical decision, which should be based on what is most useful for the decision-making context.

This review found that most ID guidelines focused on technical aspects, including selection of appropriate model structure, choice of model parameters, and examination of uncertainty,^71,72,81^ but included limited discussion of the role of social determinants beyond characterizing disease dynamics and model predictions. When social determinants were discussed as an overarching principle of health equity, there were limited details on the specific steps to be taken to incorporate them in model conceptualization and technical implementation.

There are potential explanations for the limited focus on social determinants of health in ID modeling guidelines. Martins et al.^82^ noted that empirical analyses involving social determinants have traditionally taken a retrospective approach, evaluating current and historic association and trends, rather than predicting future scenarios and policy impact. Another potential reason, in some cases, is the lack of an evidence-informed mechanistic description of the interaction between social determinants and health outcomes to inform ID models.^67^ Modeling approaches may also be driven by the demand side; that is, decision makers may be more interested in ID models that can inform whole population-level policy options, particularly at the time when disease transmission is still being understood. Finally, data-related and computational challenges of incorporating social heterogeneity in ID models may explain the limited focus of guidelines on social determinants.

Another gap in ID guidelines relates to the interaction between public health interventions and broader financial and social policies implemented during an epidemic/pandemic. For instance, the preexisting financial indebtedness of households and societies is often exacerbated by lockdowns, interest rate changes, real estate bubbles, and devaluation of investment portfolios, resulting in jobs losses and changes in housing affordability.^83,84^ This phenomenon, sometimes referred to as “financialization” of society, affects social groups inequitably and results in an increase in shared housing arrangements in multioccupation properties, wage reductions that induce individuals to take extra jobs, or delay in the retirement or return to work of vulnerable elderly people.^85^ These changes result in an inequitable increase in the risk of infection and poor health outcomes. To address this, modeling guidelines should offer direction on integrating the broader context of inequitable financial and social changes during a pandemic that compound the underlying socioeconomic and rural/urban disparities in exposure to infection and access to health services. Our study has a number of strengths. We followed the structured approach of a systematic review, with screening and data extraction done by 2 independent researchers. We identified guidelines published since year 2000 and did not limit them by the type of ID. In addition to identifying common social determinants discussed in guidelines, we identified key recommendations to support future modeling studies. Finally, we contextualized the recommendation in relation to the current COVID-19 pandemic to inform current modeling practices.

### Limitations

Our study has a few limitations. First, we did not review gray literature, including preprint repositories and government reports, to identify non–peer-reviewed papers and reports. While this is a limitation, we made a substantial effort to identify any missed studies by consulting with experts in our team and hand searching references. Next, we included only studies published in English, which may have missed guidelines published in other languages. Finally, in the absence of a standard quality checklist for modeling guidelines, we used relevant domains of a clinical guidelines quality checklist. However, we did not exclude studies based on the quality appraisal; this approach allowed us to identify recommendations using a larger pool of studies.

## Conclusion

The World Health Organization has highlighted the need to take appropriate account of social determinants of health in pandemic response efforts, including predicting the course of the pandemic, developing policy interventions, and managing vaccine programs.^86^ The need has become more urgent as the COVID-19 pandemic has exacerbated existing health inequities and significantly affected marginalized groups.^50,62,87,88^ The pandemic has revived attention on social determinants in the modeling community. However, only a limited number of studies have incorporated them in ID models.^89–91^ Progress is hampered by lack of clear guidance on how to consider social determinants in modeling. Our systematic review makes a contribution to bridge this gap.

This systematic review has highlighted that ID modeling should not be considered a mathematical exercise but rather a public health tool to support decision making in the real world. In this sense, understanding social heterogeneity and disparity of disease risk and burden to inform policy action is the most important role of ID models. Without such consideration, ID models have the potential to increase disparities by ignoring the distributional impact of policies across social groups.

## Supplemental Material

sj-pdf-1-mdm-10.1177_0272989X241280611 – Supplemental material for Incorporating Social Determinants of Health in Infectious Disease Models: A Systematic Review of Guidelines Supplemental material, sj-pdf-1-mdm-10.1177_0272989X241280611 for Incorporating Social Determinants of Health in Infectious Disease Models: A Systematic Review of Guidelines by Shehzad Ali, Zhe Li, Nasheed Moqueet, Seyed M. Moghadas, Alison P. Galvani, Lisa A. Cooper, Saverio Stranges, Margaret Haworth-Brockman, Andrew D. Pinto, Miqdad Asaria, David Champredon, Darren Hamilton, Marc Moulin and Ava A. John-Baptiste in Medical Decision Making
