# Supplementary material for: Incorporating Social Determinants of Health in Infectious Disease Models: A Systematic Review of Guidelines
Source: Med Decis Making. 2024 Sep 21;44(7):742–55. doi: 10.1177/0272989X241280611 (PMC11491037; doi:10.1177/0272989X241280611)
Supplement: sj-pdf-1-mdm-10.1177_0272989X241280611 – Supplemental material for Incorporating Social Determinants of Health in Infectious Disease Models: A Systematic Review of Guidelines [file sj-pdf-1-mdm-10.1177_0272989X241280611.pdf]

Appendix S1

Search strategy to identify guidelines for infectious disease modeling

Database: Embase Classic+Embase <1947 to 2020 September 10>

Search Strategy:

----

1. Theoretical Model/ (90145)
2. Markov Chain/ or stochastic.ti,ab,kw. (44451)
3. (forecast* or model* or simulat*).ti,ab,kw. (4094081)
4. Computer Simulation/ (119308)
5. Monte Carlo method/ (40806)
6. (Math* adj3 model*).ti,ab,kw. (71858)
7. (markov chain* or markov process*).ti,ab,kw. (8361)
8. "monte carlo method".ti,ab,kw. (3370)
9. (computer simulation or computer* model*).ti,ab,kw. (26932)
10. transmi*.ti,ab,kw. (617226)
11. Transmission dynamic*.ti,ab,kw. (4212)
12. ((transmission or outbreak* or epidemi* or compartmental or deterministic or individual-based or agent-based or network* or prediction* or infectious disease* or markov or dynamic* or simulation or patient specific) adj3 model*).ti,ab,kw. (180626)
13. ((data or contact or dynamic* or social or sensor or transmission) adj3 network*).ti,ab,kw. (49365)
14. or/1-13 (4712650)
15. (guideline* or guidance).ti,ab,kw. or Practice Guideline/ (915281)
16. Communicable Disease/ (30710)
17. Disease Transmission/ (99705)
18. or/16-17 (128464)
19. 14 and 15 and 18 (2473)
20. limit 19 to (embase and yr="2000 -Current") (1395)

Appendix S2

List of identified guidelines for infectious disease modeling

1. Abuelezam NN, Rough K, Seage III GR (2013) Individual-Based Simulation Models of HIV Transmission: Reporting Quality and Recommendations. PLoS ONE 8(9): e75624. doi:10.1371/journal.pone.0075624
2. Andradóttir, S., Chiu, W., Goldsman, D., & Lee, M. L. (2014). Simulation of influenza propagation: Model development, parameter estimation, and mitigation strategies. IIE Transactions on Healthcare Systems Engineering, 4(1), 27-48.
3. Barnes SL, Kasaie P, Anderson DJ, Rubin M. Research Methods in Healthcare Epidemiology and Antimicrobial Stewardship - Mathematical Modeling. Infection Control and Hospital Epidemiology. 2016;37(11):1265-71.
4. Behrend, M. R., Basáñez, M. G., Hamley, J. I., Porco, T. C., Stolk, W. A., Walker, M., ... & NTD Modelling Consortium. (2020). Modelling for policy: The five principles of the Neglected Tropical Diseases Modelling Consortium. PLoS neglected tropical diseases, 14(4), e0008033.
5. Caro JJ, Briggs AH, Siebert U, Kuntz KM. Modeling good research practices - Overview: A report of the ISPOR-SMDM modeling good research practices task force-1. Value in Health. 2012;15(6):796-803.
6. Caro J, Eddy DM, Kan H, Kaltz C, Patel B, Eldessouki R, Briggs AH; ISPOR-AMCP-NPC Modeling CER Task Forces. Questionnaire to assess relevance and credibility of modeling studies for informing health care decision making: an ISPOR-AMCP-NPC Good Practice Task Force report. Value Health. 2014 Mar;17(2):174-82. doi: 10.1016/j.jval.2014.01.003. Erratum in: Value Health. 2016 Jan;19(1):121.
7. Collaborating Group on Chagas Disease Modelling (CGCDM). Insights from quantitative and mathematical modelling on the proposed WHO 2030 goals for Chagas disease. Gates Open Research. 2019 Sep 17;3:1539. https://gatesopenresearch.org/articles/3-1539
8. COVID-19 Multi-Model Comparison Collaboration (CMCC) Policy Group. Guidance on Use of Modelling for Policy Responses to COVID-19. (conference proceeding); 2020
9. den Boon S, Jit M, Brisson M, Medley G, Beutels P, White R, et al. Guidelines for multi-model comparisons of the impact of infectious disease interventions. BMC medicine. 2019;17(1):163.
10. Drake T, Medley G, Vassall A, Gomez G. International Decision Support Initiative (IDSI). Equity, economic evaluation, and disease transmission modelling–26-27th March 2018: Pre-meeting reviews. F1000Research. 2019 Jun 20. DOI: https://doi.org/10.7490/f1000research.1116870.1

11. Funk, S., Bansal, S., Bauch, C. T., Eames, K. T., Edmunds, W. J., Galvani, A. P., & Klepac, P. (2015). Nine challenges in incorporating the dynamics of behaviour in infectious diseases models. Epidemics, 10, 21-25.
12. Jit, M., & Brisson, M. (2011). Modelling the epidemiology of infectious diseases for decision analysis. Pharmacoeconomics, 29(5), 371-386.
13. Knight, G. M., Dharan, N. J., Fox, G. J., Stennis, N., Zwerling, A., Khurana, R., & Dowdy, D. W. (2016). Bridging the gap between evidence and policy for infectious diseases: How models can aid public health decision-making. International journal of infectious diseases, 42, 17-23.
14. Moghadas SM, Haworth-Brockman M, Isfeld-Kiely H, Kettner J. Improving public health policy through infection transmission modelling: Guidelines for creating a community of practice. Canadian Journal of Infectious Diseases and Medical Microbiology. 2015;26(4):191-5.
15. Pitman R, Fisman D, Zaric GS, Postma M, Kretzschmar M, Edmunds J, et al. Dynamic transmission modeling: a report of the ISPOR-SMDM Modeling Good Research Practices Task Force--5. Value in health : the journal of the International Society for Pharmacoeconomics and Outcomes Research. 2012;15(6):828-34.
16. Roberts, M., Russell, L. B., Paltiel, A. D., Chambers, M., McEwan, P., & Krahn, M. (2012). Conceptualizing a model: a report of the ISPOR-SMDM modeling good research practices task force–2. Medical Decision Making, 32(5), 678-689.
17. Russell RE, Katz RA, Richgels KLD, Walsh DP, Grant EHC. A Framework for Modeling Emerging Diseases to Inform Management. Emerging infectious diseases. 2017;23(1):1-6.
18. Ultsch B, Damm O, Beutels P, Bilcke J, Bruggenjurgen B, Gerber-Grote A, et al. Methods for Health Economic Evaluation of Vaccines and Immunization Decision Frameworks: A Consensus Framework from a European Vaccine Economics Community. Pharmacoeconomics. 2016;34(3):227-44.
19. Woolhouse M. How to make predictions about future infectious disease risks. Philosophical Transactions of the Royal Society B-Biological Sciences. 2011;366(1573):2045-54.

Appendix Table S1. Results of critical appraisal.

Author, year	Domain 1	Domain 2	Domain 3	Domain 4	Domain 5	Domain 6
Abuelezam, 2013	100.0%	75.0%	70.8%	95.8%	88.9%	91.7%
Andradottir, 2014	75.0%	62.5%	45.8%	54.2%	63.9%	83.3%
Barnes, 2016	83.3%	58.3%	16.7%	91.7%	86.1%	95.8%
Behrend, 2020	91.7%	91.7%	70.8%	91.7%	88.9%	91.7%
Caro, 2012	100.0%	95.8%	87.5%	95.8%	91.7%	45.8%
Caro, 2014	91.7%	95.8%	87.5%	91.7%	88.9%	87.5%
CGCDM, 2019	66.7%	62.5%	37.5%	70.8%	75.0%	87.5%
CMCC, 2020	83.3%	66.7%	45.8%	83.3%	80.6%	87.5%
Den Boon, 2019	91.7%	91.7%	79.2%	91.7%	94.4%	91.7%
Drake, 2018	83.3%	66.7%	37.5%	79.2%	58.3%	79.2%
Funk, 2015	83.3%	54.2%	20.8%	83.3%	83.3%	75.0%
Jit, 2011	91.7%	45.8%	70.8%	91.7%	83.3%	95.8%
Knight, 2016	91.7%	70.8%	29.2%	91.7%	88.9%	91.7%
Moghadas, 2015	83.3%	70.8%	37.5%	83.3%	75.0%	91.7%
Pitman, 2012	83.3%	70.8%	79.2%	91.7%	91.7%	58.3%
Roberts, 2012	91.7%	83.3%	87.5%	91.7%	86.1%	79.2%
Russel, 2017	66.7%	66.7%	29.2%	87.5%	80.6%	70.8%
Ultsch, 2016	83.3%	70.8%	83.3%	87.5%	86.1%	75.0%
Woolhouse, 2011	83.3%	25.0%	54.2%	66.7%	77.8%	83.3%
